# Readability of the 100 Most-Cited Neuroimaging Papers Assessed by Common Readability Formulae

**DOI:** 10.3389/fnhum.2018.00308

**Published:** 2018-08-14

**Authors:** Andy W. K. Yeung, Tazuko K. Goto, W. Keung Leung

**Affiliations:** ^1^Oral and Maxillofacial Radiology, Applied Oral Sciences, Faculty of Dentistry, The University of Hong Kong, Hong Kong, Hong Kong; ^2^Department of Oral and Maxillofacial Radiology, Tokyo Dental College, Tokyo, Japan; ^3^Periodontology, Faculty of Dentistry, The University of Hong Kong, Hong Kong, Hong Kong

**Keywords:** bibliometrics, information science, neuroimaging, neurosciences, readability

## Abstract

**Background:** From time to time, neuroimaging research findings receive press coverage and attention by the general public. Scientific articles therefore should be written in a readable manner to facilitate knowledge translation and dissemination. However, no published readability report on neuroimaging articles like those published in education, medical and marketing journals is available. As a start, this study therefore aimed to evaluate the readability of the most-cited neuroimaging articles.

**Methods:** The 100 most-cited articles in neuroimaging identified in a recent study by Kim et al. (2016) were evaluated. Headings, mathematical equations, tables, figures, footnotes, appendices, and reference lists were trimmed from the articles. The rest was processed for number of characters, words and sentences. Five readability indices that indicate the school grade appropriate for that reading difficulty (Automated Readability Index, Coleman-Liau Index, Flesch-Kincaid Grade Level, Gunning Fog index and Simple Measure of Gobbledygook index) were computed. An average reading grade level (AGL) was calculated by taking the mean of these five indices. The Flesch Reading Ease (FRE) score was also computed. The readability of the trimmed abstracts and full texts was evaluated against number of authors, country of corresponding author, total citation count, normalized citation count, article type, publication year, impact factor of the year published and type of journal.

**Results:** Mean AGL ± standard deviation (SD) of the trimmed abstracts and full texts were 17.15 ± 2.81 (college graduate level) and 14.22 ± 1.66 (college level) respectively. Mean FRE score ± SD of the abstracts and full texts were 15.70 ± 14.11 (college graduate level) and 32.11 ± 8.56 (college level) respectively. Both items indicated that the full texts were significantly more readable than the abstracts (*p* < 0.001). Abstract readability was not associated with any factors under investigation. ANCOVAs showed that review/meta-analysis (mean AGL ± SD: 16.0 ± 1.4) and higher impact factor significantly associated with lower readability of the trimmed full texts surveyed.

**Conclusion:** Concerning the 100 most-cited articles in neuroimaging, the full text appears to be more readable than the abstracts. Experimental articles and methodology papers were more readable than reviews/meta-analyses. Articles published in journals with higher impact factors were less readable.

## Introduction

Neuroimaging research field frequently witnessed advancements in knowledge, understanding and technology. Nowadays, scientists have utilized neuroimaging to understand and treat brain disorders such as depression, and to predict behavior which is relevant to marketing and policy-making (Poldrack and Farah, 2015). Experimental papers provide knowledge from basic science that enhances fundamental understanding of any biology involved, and from clinical science that contributes to evidence-based clinical practices. For example, brain connectivity at resting state discovered two decades ago (Biswal et al., 1995) is now having a potential to identify patients with Alzheimer’s disease (de Vos et al., 2018). Methodological papers introduce novel ways to acquire and analyze neuroimaging data that may contribute to the understanding of normal neurophysiology, clinical diagnostics or therapeutic approaches. For instance, the voxel-based morphometry method developed to detect brain structural changes (Ashburner and Friston, 2000) is now applied to study such subtle changes in Parkinson’s disease patients with early cognitive impairment (Gao et al., 2017). Systematic reviews and meta-analyses also very important for pooling evidence across studies to discuss and evaluate whether specific findings are consistently reported, and thus particular theories or therapeutic strategy could be established or rejected (Yeung et al., 2017b,d, 2018a). With such a diverse literature, bibliometric reports have an important value because they quantitatively and qualitatively evaluate the peer scientific impact of the academic literature on specific breakthroughs or selected research topics, with the analyses on publication and citation information as well as the content of the papers (Carp, 2012; Guo et al., 2014; Kim et al., 2016; Yeung, 2017a,b, 2018a,b; Yeung et al., 2018b). In particular, a bibliometric study on the readability of neuroimaging papers would be very helpful, as the literature has indicated that there exist translation or communication challenges of neuroimaging research when the findings are disseminated to the public (O’Connor et al., 2012; van Atteveldt et al., 2014).

To avoid the translation or communication challenges, neuroimaging and neuroscience articles should be written in a readable manner to better disseminate the existing knowledge and novel findings (Illes et al., 2010). From time to time, neuroimaging findings received press coverage (Herculano-Houzel, 2002; Racine et al., 2005; O’Connor et al., 2012) and influenced the society through various means such as substantiating morality claims, justifying healthcare procedures and facilitating policy making (Racine et al., 2005). A 10-year survey on mass media identified over 1,000 news articles reporting neuroimaging research findings, most of which were written by journalists (84%) and press agencies (11%) (Racine et al., 2010). Because neuroimaging affects real-world social contexts and creates news headlines, it is important for people working for the press to understand the underlying scientific findings, interpretations and limitations (Racine et al., 2005; O’Connor et al., 2012). The simplified take-home message translated from brain researches to the public should be correct and contain no misconceptions (Beck, 2010; van Atteveldt et al., 2014). Unfortunately the latter is not uncommon. Misconceptions disseminated by journalists may arise from four reasons, namely the accuracy or internal consistency of the articles themselves, articles having a firm conclusion supported by weak data, inappropriate extrapolation of basic and pre-clinical findings to therapeutic uses, and selectively reporting findings published in journals with high impact factors only (Gonon et al., 2011, 2012). Some argued that misconceptions may also arise among practitioners such as those in education because neuroscience articles were not easily understood by lay people (Howard-Jones, 2014). Meanwhile, concerns regarding readability of journal articles is potentially an universal issue as surveys have revealed that the health literacy skills, including print literacy (McCray, 2005), of the general public proficient in English were at or below the eighth grade level (Herndon et al., 2011). Therefore, healthcare journalists should dispense the biomedical research findings to the lay public with accurate interpretation and appropriate language level (Angell and Kassirer, 1994; Eggener, 1998). However, the majority of the healthcare journalists had bachelor’s degrees unrelated to healthcare and had no additional postgraduate degrees (Viswanath et al., 2008; Friedman et al., 2014). Moreover, they often lack time and knowledge to improve the informative value of their reports (Larsson et al., 2003), sometimes had difficulties in understanding the original papers (Friedman et al., 2014) and the news coverage often emphasized on the beneficial effects of the experimental treatments stated in the article abstracts from journals with high impact factors (Yavchitz et al., 2012). Results from these reports indicated an evaluation of the readability of neuroimaging articles and their abstracts is needed, which, to the best of the authors’ knowledge, has not been documented by previously published studies. To begin with, it would be beneficial to evaluate such a factor concerning the 100 neuroimaging articles that received the most all-time attention in terms of scientific citations (Kim et al., 2016). The rationale for choosing this collection of articles was that a collection of “100 most cited articles” was often perceived as a representative sample of the most influential works within the field of interest and this sampling strategy has been continuously published (Joyce et al., 2014; Powell et al., 2016; Wrafter et al., 2016). Hence, the 100 most cited articles are considered drivers for fundamental knowledge development, knowledge application and evidence-based practice with regard to their fields of interest. Moreover, research articles with high impact often received increased media attention (Gonon et al., 2011, 2012; Yavchitz et al., 2012), which underlines the importance of their readability.

Readability of academic articles may be associated with author, article and journal factors, such as the number of authors (Plavén-Sigray et al., 2017), first language of the principal author (Hayden, 2008), country of institutional affiliation of the first author (Weeks and Wallace, 2002), citation count (Gazni, 2011), publication year (Plavén-Sigray et al., 2017), article type (Hayden, 2008), and choice of journal (Shelley and Schuh, 2001; Weeks and Wallace, 2002). Editorial and peer-review process could also improve the readability of the manuscripts (Roberts et al., 1994; Rochon et al., 2002; Hayden, 2008). Based on these considerations, the aim of this study was to assess the 100 most-cited neuroimaging articles to evaluate their readability level. We hypothesized that the abstracts, as the summaries of the articles, would be more readable than the full texts. We also hypothesized that, for both abstracts and full texts, the readability of these most-cited articles published across the years would be similar.

## Materials and Methods

### Data Selection, Retrieval and Processing

The 100 most-cited articles in neuroimaging identified in a recent study (Kim et al., 2016) were evaluated. Fifty of these 100 articles were published in journals classified by Journal Citation Reports as specialized in neuroimaging, namely *NeuroImage* (*n* = 38) and *Human Brain Mapping* (*n* = 12). The other fifty articles were published in other journals, led by *Magnetic Resonance in Medicine* (*n* = 10). The full articles were individually copied into Microsoft Word and trimmed – headings/subheadings, mathematical equations, tables, figures, footnotes, appendices, and reference lists were removed, as described earlier (Sawyer et al., 2008; Jayaratne et al., 2014). To account for the differences in styles of in-text citations (e.g., superscript numbers versus lists of authors with publication year), the in-text citations were removed. Finally, there were two copies of processed text prepared for each article: abstract and full text.

As reviewed in the introduction, nine factors can potentially influence the readability of an article. Since we did not have access to manuscripts to compare their contents pre-, during and post-editorial and peer-review process, we decided to exclude this factor for the current investigation. Moreover, the authors have considered the number of tables, figures and references as potential factors to be evaluated, but decided to drop them because a study showed that these factors did not associate with readability (Weeks and Wallace, 2002). Therefore, the following eight potential influencing factors (“Factors”) were recorded for each article:

(1) Number of authors
(2) Country of the institution of the corresponding author
(3) Total citation count
(4) Normalized citation count (i.e., total count divided by years since publication)
(5) Publication year
(6) Article type (i.e., experimental article, methodology paper or review/meta-analysis)
(7) Impact factor of the publication year of the journal
(8) Type of journal (i.e., journals specialized in neuroimaging or other journals)

Regarding authors, Hayden (2008) binarized first language of the principal author into English and non-English, whereas Weeks and Wallace (2002) compared first authors working in United States and United Kingdom. We believed that, for the current study, it would be more appropriate to evaluate the country of the institutional affiliation of the corresponding author, because the corresponding author should be responsible for overseeing the work and sometimes it is difficult to determine the first language of the authors including the principal author.

The list of the 100 articles with their information on these eight Factors can be found in **Supplementary Table S1**.

### Readability Assessment

The readability statistics were computed with the Readability Calculator^1^, a free online program used by previous studies to assess the readability of Web materials (Mcinnes and Haglund, 2011; Jayaratne et al., 2014). The edited text for each article was processed by this website to count the number of characters, words and sentences. The website also computed five readability indices that indicate the school grade appropriate for that reading difficulty. The readability grade level indices were the Automated Readability Index, Coleman-Liau Index, Flesch-Kincaid Grade Level, Gunning Fog index and Simple Measure of Gobbledygook index. All these indices produce an output that approximates the grade level (in United States) estimated necessary to understand the text. Each of them use a formula that differs from each other slightly, particularly the Automated Readability Index and Coleman-Liau Index rely on character counts whereas the others rely on syllable counts. The Gunning Fog index was the most frequently used index to evaluate readability of journal articles (Roberts et al., 1994; Rochon et al., 2002; Weeks and Wallace, 2002), whereas the others were also often used to check readability of journal articles as well as materials on websites targeting patients (Sawyer et al., 2008; Jayaratne et al., 2014). An average reading grade level (AGL) was calculated by taking the mean of these five indices (Sawyer et al., 2008; Jayaratne et al., 2014). Besides the school grade indices, a Flesch Reading Ease (FRE) score was also computed for each article. This score was also frequently used to evaluate readability of journal articles (Roberts et al., 1994; Rochon et al., 2002; Weeks and Wallace, 2002; Plavén-Sigray et al., 2017). In contrast to the school grade indices, a higher FRE score means easier to read. The formulas of the abovementioned indices are listed in **Table 1**.

**Table 1:** Formulas of the readability grade level indices and Flesch Reading Ease score.

Index	Formula
Automated Readability Index	4.71 × (characters/word) + 0.5 × (words/sentence) – 21.43
Coleman-Liau Index	0.0588 × (characters/100 words) – 0.296 × (sentences/100 words) – 15.8
Flesch-Kincaid Grade Level	(0.39 × words/sentence) + (11.8 × syllables/word) – 15.59
Gunning Fog index	0.4 × (words/sentence + percentage of ≥ 3-syllable words)
Simple Measure of Gobbledygook index	3 + (square root of≥3-syllable words count/30 sentences)
Average reading grade level (AGL)	Mean value of the above five indices
	
Flesch Reading Ease (FRE) score	206.835 – (1.015 × words / sentence) – (84.6 × syllables/word)


It should be noted that these formulae assume the readability is related to the number of long words or sentences without considering the contextual difficulty of the words, such as the use of jargon. However, it is difficult to define what is jargon. There is a formula called New Dale-Chall readability formula that outputs a numerical value representing the comprehension difficulty of the surveyed text (Chall and Dale, 1995): Raw Score = 0.1579 ^∗^ (% of difficult words) + 0.0496 ^∗^ (words/sentences). It uses a list of 3,000 common words, and words outside this list are considered as difficult words. However, no published report has defined the common word list within the context of scientific journal articles, so it would be difficult to consider the effect of jargon and hence that issue was not followed in the 100 articles investigated.

### Data Analysis

Cronbach’s alpha was computed for the scores from the five readability grade level indices to evaluate their internal consistency in representing the grade level of the trimmed abstracts and full texts, respectively. A value of 0.7 or above indicated acceptable to excellent internal consistency (Bland and Altman, 1997; Tavakol and Dennick, 2011). Paired t-tests were performed to evaluate if there were significant differences in readability between the trimmed abstracts and full texts of the articles.

Univariate linear regressions were separately performed to evaluate if the abstract and full text readability (i.e., the AGL and FRE score) of the concerned articles were associated with continuous independent variable, i.e., (1) the number of authors, (2) total citation count, (3) normalized citation count, (4) publication year and (5) impact factor. One-way ANOVAs were separately performed to evaluate if there were significant associations between readability data and categorical independent variables such as (1) countries of the institutions of the corresponding authors, (2) article types, and (3) journal type (journal type was tested by two-sample *t*-tests); *post hoc* Tukey tests were conducted to reveal the significantly different pairs. Paired *t*-tests (**Table 4**) were performed to check if there were significant differences in the readability between the abstracts and full texts according to different countries, journal types, and article types.

Finally, if the above univariate tests revealed significant associations, multi-way ANCOVAs were performed to evaluate if any of these factors, when considered together, would still be associated with the readability scores. All statistical analyses were performed in SPSS 24.0 (IBM, Armonk, New York, United States). Test results were significant if *p* < 0.05.

## Results

The 100 articles were published between 1980 and 2012, among which 37 were experimental articles, 48 were methodology papers and 15 were reviews/meta-analyses. The number of authors for each article ranged from one to 21 (mean ± SD: 5.6 ± 3.5), with three-fourths of the articles having two to seven authors. The institutions of corresponding authors of 55 articles were located in the United States, followed by 27 in the United Kingdom and 18 in the rest of the world (five in Canada, four in France, three in Germany and one each in Australia, Austria, Sweden, Switzerland, Netherlands, and Denmark). Total citation count of the articles ranged from 673 to 4,384 (1,328 ± 722), whereas normalized citation count ranged from 24.9 to 313.1 (88.2 ± 59.3). For journal impact factor of the respective publication years, we could only retrieve data for 62 articles^2^. It ranged from 1.914 to 24.520 (6.577 ± 4.14). The full texts had 5,484–107,338 characters, 1,027–19,795 words, 49–871 sentences and 14.6–28.5 words per sentence (**Table 2**).

**Table 2:** Readability of the abstracts and full texts of the 100 neuroimaging articles.

Assessment item	Mean (*SD*)		*p*-value
		
	Abstracts	Full texts	
Automated Readability Index	16.77 (3.68)	15.04 (1.37)	<0.001
Coleman-Liau Index	15.89 (2.45)	13.24 (1.49)	<0.001
Flesch-Kincaid Grade Level	16.92 (3.02)	13.83 (1.70)	<0.001
Gunning Fog index	18.98 (3.34)	15.84 (1.85)	<0.001
Simple Measure of Gobbledygook index	17.19 (2.34)	15.04 (1.37)	<0.001
Average reading grade level (AGL)^a^	17.15 (2.81)	14.22 (1.66)	<0.001
No. of characters	1,084 (382)	30,212 (16,949)	N/A
No. of words	194 (69)	5,780 (3,113)	N/A
No. of sentences	8.88 (4.86)	287.00 (149.51)	N/A
No. of words/sentence	23.56 (5.83)	20.31 (2.74)	<0.001
Flesch Reading Ease (FRE) Score^b^	15.70 (14.11)	32.11 (8.56)	<0.001


*Paired *t*-tests were performed for all assessment items. ^*a*^AGL: ≤12 is high school level or below, 13–16 is college level, >16 is college graduate level. ^*b*^FRE scores: <30 is college graduate level, 30–50 is college level, >50 is high school level or below.*

### Internal Consistency of the Five Readability Grade Level Indices

The five indices demonstrated excellent internal consistency in assessing the trimmed abstracts (α = 0.96) and full texts (α = 0.98) of the 100 articles. Therefore, an AGL score was representative to be used for subsequent analyses.

### Differences Between Abstracts and Full Texts

The detailed readability assessment results of the trimmed abstracts and full texts are listed in **Table 2**. The AGL of the trimmed abstracts and full texts corresponded with the grade of college graduates and college sophomores, respectively. The difference was significant (*t* = 11.64, *p* < 0.001), indicating that the abstracts were less readable.

**FIGURE 1:**
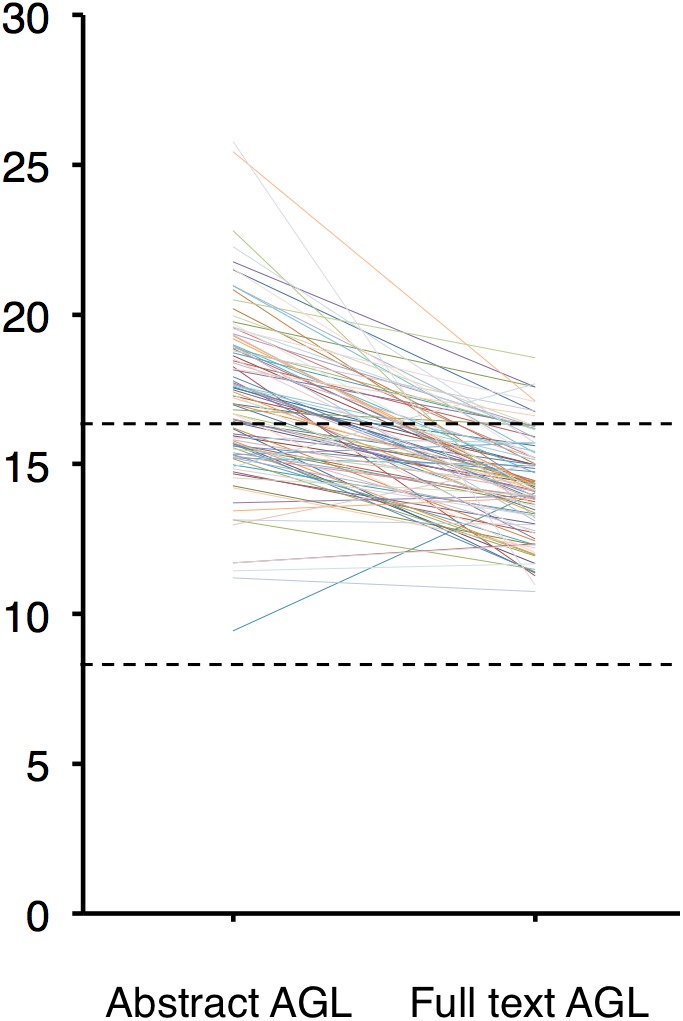
Average reading grade level (AGL) of the 100 abstracts and their corresponding full texts. None of them had AGL < 8 (lower dotted line), the readability level of the general public. Thirty-seven abstracts and 84 full texts had AGL < 16 (upper dotted line), i.e., below college graduate level.

The AGL of each of the trimmed abstracts and their corresponding full texts were plotted (**Figure 1**): 37 abstracts and 84 full texts had an AGL < 16, i.e., below college graduate level. All these 37 abstracts except one had their corresponding full text AGL < 16. The exception had a full text AGL of 17.70. No abstract or full text had an AGL < 8, i.e., general public level.

The FRE scores of the trimmed abstracts and full texts (**Table 2**) were considered “very difficult” and “difficult,” respectively (Flesch, 1948). The difference was significant (*t* = -13.30, *p* < 0.001), again indicating that the abstracts were less readable.

### Readability of Articles Evaluated by Univariate Analyses

The number of authors (Factor 1), total citation count (Factor 3) and normalized citation count (Factor 4) had no significant association with the readability of the trimmed abstracts and full texts of the concerned articles (**Table 3**).

**Table 3:** Results from univariate linear regressions.

	Abstracts				Full texts			
		
Factors	AGL (*r*^2^)	*p*-value	FRE score (*r*^2^)	*p*-value	AGL (*r*^2^)	*p*-value	FRE score (*r*^2^)	*p*-value
Author number	0.030	0.083	0.029	0.092	0.004	0.529	0.006	0.439
Total citation count	0.007	0.400	0.011	0.289	0.005	0.470	0.008	0.373
Normalized citation count	0.000	0.876	0.002	0.690	0.006	0.447	0.003	0.577
Publication year	0.023	0.131	0.016	0.208	0.066	0.010	0.053	0.021
Impact factor	0.007	0.525	0.003	0.677	0.114	0.007	0.087	0.020


**r*^2^, coefficient of determination. AGL, Average reading grade level. FRE, Flesch Reading Ease.*

The country of the institution of the corresponding author (Factor 2) had no significant association with the readability of the trimmed abstracts and full texts (**Table 4**). No statistical test was attempted to compare corresponding authors from English-speaking countries (i.e., United States, United Kingdom, Canada, and Australia, *n* = 88) versus non-English-speaking countries (*n* = 12).

**Table 4:** Results from one-way ANOVA and two-sample *t*-tests.

	Readability assessment			
	
	Abstracts		Full texts	
		
Factors	AGL^a^	FRE score^b^	AGL^a^	FRE score^b^
Country			
United States	17.0 (2.7)	17.0 (12.8)	14.4 (1.6)^∗∗^	30.8 (7.9)^∗∗^
United Kingdom	17.3 (2.3)	14.9 (12.8)	13.9 (1.6)^∗∗^	34.3 (8.0)^∗∗^
Rest of the world	17.5 (3.7)	12.9 (19.4)	14.1 (2.0)^∗^	32.7 (10.7)^∗∗^
	*p* = 0.779	*p* = 0.535	*p* = 0.338	*p* = 0.209
			
Article type			
Experimental articles	16.6 (3.3)	17.8 (16.7)	14.0 (1.8)^∗∗^	32.6 (8.7)^∗∗^
Methodology papers	17.1 (2.3)	16.1 (11.5)	13.8 (1.3)^∗∗^	34.5 (7.0)^∗∗^
Reviews/meta-analyses	18.5 (2.7)	9.1 (13.7)	16.0 (1.4)^∗^	23.2 (7.4)^∗^
	*p* = 0.085	*p* = 0.121	*p* < 0.001	*p* < 0.001
			
Journal type			
Neuroimaging	17.6 (2.6)	13.7 (13.1)	14.2 (1.6)^∗∗^	31.8 (8.9)^∗∗^
Others	16.7 (3.0)	17.8 (14.9)	14.2 (1.8)^∗∗^	32.4 (8.3)^∗∗^
	*p* = 0.103	*p* = 0.143	*p* = 0.864	*p* = 0.727


*Standard deviations in parentheses. Supplementary paired *t*-tests for full text versus abstracts: ^∗^*p* < 0.01, ^∗∗^*p* < 0.001. ^*a*^AGL, Average reading grade level: ≤12 is high school level or below, 13–16 is college level, >16 is college graduate level. ^*b*^FRE, Flesch Reading Ease scores: <30 is college graduate level, 30–50 is college level, >50 is high school level or below.*

Publication year (Factor 5) had no significant association with the AGL or FRE score for trimmed abstracts (**Table 3**). For trimmed full texts, the more recent publications appear to have a higher AGL and a lower FRE score, meaning that they are less readable.

Article type (Factor 6) had no significant association with the AGL or FRE score for trimmed abstracts (**Table 4**). For trimmed full texts, reviews/meta-analyses were significantly less readable than experimental articles and methodology papers, with AGL/FRE scores of 16.0/23.2, 14.0/32.6 and 13.8/34.5 respectively (*p* < 0.001). These results imply that the readability of reviews/meta-analyses were at college graduate level whereas that of experimental and methodology papers were at college level.

Impact factor (Factor 7) had no significant association with the AGL or FRE score for trimmed abstracts (**Table 3**). For trimmed full texts published after 1996, impact factor appears to be associated with a higher AGL (*r*^2^ = 0.114, *p* = 0.007) and a lower FRE score (*r*^2^ = 0.087, *p* = 0.020). This means that articles with higher impact factors are less readable.

Journal type (Factor 8) had no significant association with the readability of the trimmed abstracts and full texts (**Table 4**).

### Multi-Way ANCOVA Results

Because the above univariate tests demonstrated that readability of the trimmed full texts was associated with publication year (Factor 5), article type (Factor 6) and impact factor (Factor 7), these three factors were entered into multi-way ANCOVAs for trimmed full text AGL and FRE score, respectively, to investigate if these associations were still significant under multivariate analysis. Results indicated that AGL (**Table 5**) was significantly associated with article type (Factor 6) and impact factor (Factor 7), whereas FRE score (**Table 6**) was significantly associated with article type (Factor 6). In brief, the readability of experimental articles and methodology papers was two grade levels easier than that of reviews/meta-analyses. Moreover, reading grade level increased by 0.100 for every increment in impact factor by 1.

**Table 5:** Relationship between full text average reading grade level and the independent variables in the ANCOVA model.

Independent variables	Estimates	*SE*	*p*-value	Multiple
				comparison
Publication year	-0.037	0.058	0.531	
Impact factor	0.100	0.042	0.020	
Article type			<0.001	(1) = (2) < (3)
Experimental articles (1)	-2.017	0.488		
Methodology papers (2)	-2.068	0.444		
Reviews/meta-analyses (3)	0			
Intercept	89.179	116.714	0.448	


**Table 6:** Relationship between full text Flesch Reading Ease score and the independent variables in the final ANCOVA model.

Independent variables	Estimates	*SE*	*p*-value	Multiple
				comparison
Publication year	0.212	0.319	0.508	
Impact factor	-0.438	0.229	0.061	
Article type			<0.001	(1) = (2) > (3)
Experimental articles (1)	9.240	2.672		
Methodology papers (2)	10.325	2.434		
Reviews/meta-analyses (3)	0			
Intercept	-399.926	639.094	0.534	


## Discussion

To the authors’ knowledge, this was the first study to evaluate the readability of neuroimaging articles. The 100 most-cited neuroimaging articles were collectively assessed for their readability. We confirmed the second hypothesis by showing that the publication year had no significant effect on the article readability. However, the first hypothesis has to be rejected because trimmed full texts were significantly more readable than abstracts. Due to the large variations in the backgrounds of the 100 papers of interests, we were able to identify factors which might associate with readability. However, readers should be aware that the current result indicated associations while no cause-and-effect relationship could be established.

Experimental articles and methodology papers were more readable than reviews/meta-analyses. Articles published after 1996 in journals with higher impact factors appeared to be less readable in terms of AGL.

### Comparison of Readability of Scientific Papers From Other Research Fields

A survey of articles published in five leading peer-reviewed general medical journals reported an average FRE score of 15.4 (college graduate level) (Rochon et al., 2002). Similarly, surveys on articles published in *Annals of Internal Medicine* and *British Journal of Surgery* reported average FRE scores of 29.1 and 23.8, respectively (college graduate level) (Roberts et al., 1994; Hayden, 2008). All these results (FRE score < 30) imply that the surveyed medical articles were as difficult to read as legal contracts (Roberts et al., 1994; Weeks and Wallace, 2002). The average FRE score of the 100 most-cited neuroimaging papers was 32.1 (college level), indicating that the surveyed articles could be considered potentially more readable than medical articles (**Table 2**, footnote). Notwithstanding, they were still considered as difficult as reading corporate annual reports (Roberts et al., 1994). Moreover, these 100 neuroimaging papers were less readable than papers in marketing journals (average FRE score of 35.3, college level) (Sawyer et al., 2008), but more readable than papers in *PLoS One* (FRE scores mostly of 20–30, college graduate level) (Plavén-Sigray et al., 2017).

### Relevance of Results in Relation to Healthcare Journalists

No abstract or trimmed full text had AGL < 8, suggesting that the general public can directly read none of them. This highlighted the relevance of the readability of neuroimaging articles to health journalists. It was recommended that researchers should try to write the articles in a more readable way because the overall accuracy of reporting of neuroimaging articles in the newspapers is low, with minimal details (Illes et al., 2010; van Atteveldt et al., 2014). For instance, it is common for the public to believe that we use only 10% of the brain (Herculano-Houzel, 2002). Such misconceptions should not be created from the media, especially digital or social media coverage. Because results from the current study revealed that reviews/meta-analyses were less readable, extra caution should be given when these papers are simplified and reported in mass media. Previous surveys reported that the majority of healthcare journalists had a bachelor’s degree without a postgraduate degree, which was equivalent to a U.S. grade level of 16 (Viswanath et al., 2008; Friedman et al., 2014). The results from the current study indicated that the mean AGL of the 100 most-cited neuroimaging articles was 17.15th grade for abstracts and 14.22th grade for full texts. Precisely, 37 abstracts and 84 full texts had AGL < 16. In other words, the readability of the majority of the abstracts was at the college graduate level, consistent with the previous large-scale survey on biomedical and life science article abstracts (Plavén-Sigray et al., 2017). There is definitely a need to improve the readability of abstracts. Health journalists should be able to comprehend the average full texts, though they might have some difficulties in comprehending some of the average abstracts. Perhaps health journalists need to consider reading the full texts to produce more accurate media coverage, unless the abstracts published in the future are more readable. This is consistent with the finding that half of the healthcare journalists wanted tighter communications with the researchers to report their studies (Friedman et al., 2014).

### Trimmed Full Texts Were More Readable Than Abstracts

Journal articles are one of the most important knowledge sources for academics, graduate and university/college students. According to a recent survey, they are the most frequently used primary resource type for research, while half of the surveyed researchers made annotations on fewer than one-third of their article collections only (Hemminger et al., 2007). That phenomenon is yet to be clarified; for instance, was that due to poor readability of the unannotated articles? Future surveys may try to find out the average reading grade of researchers and scientists working in the neuroimaging field because this information is currently lacking. Healthcare and life science journals such as the *Proceedings of the National Academy of Sciences* and *PLoS Medicine* have adopted the practice of using plain language summaries of research to disseminate research findings to a broader audience of the general public, students, researchers and journalists (Shailes, 2017). The booming neuroimaging literature (Yeung et al., 2017a,c,e) certainly requires accurate, sufficient yet understandable accounts of the research performed to translate and disseminate the new knowledge gained (Illes et al., 2010). However, the current study revealed that full texts were significantly more readable than abstracts. This implied that scientific report authors should compose more readable abstracts, or the journal editors might demand such abstracts or edit the abstracts to make them more readable. It is imperative to make abstracts more readable as they are often what journalists read and serve as the basis to write news coverage (Gonon et al., 2011; Yavchitz et al., 2012). Alternatively, interested researchers and medical journalists might have to read the full text for a better comprehension, which is often not practical if time does not allow.

The number of authors was not significantly associated with the readability of the articles. It was reasonable because we expect first and/or corresponding authors would do most of the writings instead of all authors. Moreover, researchers including those in the neuroimaging field should have high literacy levels and all these 100 most-cited neuroimaging articles were published in reputable journals. Therefore, it was not surprising to find that total citation count, normalized citation count, country of the institution of the corresponding author and journal type were not significantly associated with the readability of the articles.

### Experimental Articles and Methodology Papers Were More Readable Than Reviews/Meta-Analyses

We were able to replicate the findings in the field of surgery that reviews and meta-analyses were less readable than experimental articles (Hayden, 2008). We are unsure about the reason behind this. However, this is a critical phenomenon: reviews and meta-analyses summarize the evidence from the existing literature, clinical trials and others and therefore they are very informative. The referees and editors could effectively guide the authors to make the manuscript more readable (Remus, 1980; Roberts et al., 1994), for instance below 16th grade level to match the academic background of healthcare journalists (Viswanath et al., 2008; Friedman et al., 2014). On the other hand, methodology papers explain procedures and rationale behind and these will potentially be followed by a massive number of fellow researchers. Therefore, it is also important for them to be readable.

### Articles Published in Journals With Higher Impact Factors Appeared to Be Less Readable

There have been very limited research reports on the relationship of article readability to journal impact factor or article type. Previously, it was reported that abstract readability was not affected by journal impact factor within the fields of biology/biochemistry, chemistry, and social sciences (Didegah and Thelwall, 2013). In the current study, we were able to replicate their findings. However, concerning top cited neuroimaging articles, trimmed full texts in journals with higher impact factors appeared to be less readable in terms of AGL. This was not too surprising, as the papers published in prestigious medical journals with high impact factors, such as *British Medical Journal* and *Journal of the American Medical Association*, were also “extremely difficult to read” as evaluated by similar readability scores (Weeks and Wallace, 2002). Besides, high impact factor journals may have a compact format. For example, one of the surveyed articles was published in Nature Neuroscience (impact factor in 2003 = 15.1), which requires 2,000–4,000 words for main text with no more than eight figures and/or tables. The implication is that the editorial boards of the top journals with high impact factor might consider helping improve the readability of the abstracts and texts for better understanding of the readers, especially the reviews and meta-analyses. This is considered important as research reports from high impact journals often received more media attention (Gonon et al., 2011, 2012; Yavchitz et al., 2012), which highlights the importance of their readability to reduce the translational challenges.

### Study Limitations

The sample size of the current study was comparable to other readability surveys (Roberts et al., 1994; Weeks and Wallace, 2002; Hayden, 2008; Sawyer et al., 2008), but the current sample could only represent articles with considerable citation counts, hence the highest academic merits. The reason for choosing this cohort was that these most-cited articles were among the most influential ones that could potentially reach a broad audience. Readers should be aware that the chosen papers to be evaluated in the current study have been published over a wide time period (33 years), of different types, with variable number of authors, citation counts and impact factors. However, it is indeed with this diverse background hence a correlation analysis become possible, we were then able to ask what factors could be associated with better readability. We subsequently performed multi-way ANCOVA to better adjust for all these variability, instead of applying separate statistical analyses on individual factors.

This study provided the first insights into the readability of highly cited neuroimaging articles. Future studies are needed to further investigate if articles that have received less attention also exhibit similar properties. It should be noted that the readability assessment utilized linguistic formulas but did not account for readers’ knowledge background. For instance, words with many syllables or long sentences might increase the grade level scores but might not cause as much difficulty to the readers as jargon. Moreover, it could be a limitation that readability was only computed automatically. We did not evaluate the actual depth of understanding and hence the level of potential misconceptions by journalists on these 100 articles. Future studies should ask a cohort of journalists to rate the readability of a selection of articles and evaluate if they can properly conceptualize the messages the authors tried to convey, and to assess the potential relationship between poor readability and occurrence of misconceptions. Another limitation was that we were able to retrieve the impact factor data for 62 out of the 100 papers only. Moreover, we did not evaluate if the articles had strong conclusions based on weak data, one potential source of misconceptions besides readability.

## Conclusion

In conclusion, among the 100 most-cited neuroimaging articles, trimmed full texts appeared to be more readable than abstracts. Experimental articles and methodology papers were more readable than reviews/meta-analyses. Articles published after 1996 in journals with higher impact factors appeared less readable in terms of AGL. Neuroimaging researchers may consider writing more readable papers so that the medical journalists and hence the general public could better comprehend, provided that their papers are intended to reach a broader audience.

## Author Contributions

AY conceived the work, acquired and analyzed the data, and drafted the work. TG and WL facilitated the acquisition of data and critically revised the work. All authors approved the final content of the manuscript.

## Conflict of Interest Statement

The authors declare that the research was conducted in the absence of any commercial or financial relationships that could be construed as a potential conflict of interest.

## Footnotes

1 https://www.online-utility.org/

2 Web of Science (WoS) provides impact factor data from 1997, but 32 articles were published before that year. Furthermore, six articles were published after 1997, but their respective journals did not have impact factor data recorded in WoS by the time of their publication.

## Supplementary Material

TABLE S1 List of the 100 used articles including the corresponding data collected.
